# A survey of antibiotic resistance patterns among Group A Streptococcus isolated from invasive and non-invasive infections in Cape Town, South Africa

**DOI:** 10.1016/j.heliyon.2024.e33694

**Published:** 2024-06-26

**Authors:** Kimona Rampersadh, Kelin C. Engel, Mark E. Engel, Clinton Moodley

**Affiliations:** a AFRO*Strep* Research Initiative & PROTEA/Cape Heart Institute., University of Cape Town, Department of Medicine, Observatory, South Africa; b SA Cochrane Centre, South Africa Medical Research Council, Francie van Zijl Dr, Parow Valley 7501, South Africa; c Department of Pathology, Division of Medical Microbiology, Faculty of Health Sciences, University of Cape Town, Cape Town, South Africa; d The National Health Laboratory Service, Microbiology, Groote Schuur Hospital, Cape Town, South Africa

## Abstract

**Background:**

There is concern regarding the increasing resistance of Group A streptococcus (GAS) to routinely used antibiotics. GAS is a common cause of bacterial pharyngitis and more severe invasive infections such as septicaemia. Furthermore, GAS pharyngitis is the antecedent for serious conditions such as rheumatic fever and rheumatic heart disease. The study aimed to determine the antimicrobial susceptibility patterns of GAS cultured from patients with invasive and non-invasive infections from Cape Town, as part of the AFRO*Strep* Registry.

**Methods:**

Samples were provided by the AFRO*Strep* Registry, a continental endeavour aiming to document Streptococcus pyogenes infection in Africa and create the first biorepository of its kind. Ninety-five GAS isolates (invasive, n = 40; non-invasive, n = 55) were evaluated for resistance to a panel of 20 antibiotics using the Sensititre® STP6F system with MICs interpreted by CLSI break points.

**Results:**

Amongst all isolates, highest levels of resistance were observed with respect to tetracycline (8.33 %), followed by azithromycin (1.04 %) and erythromycin (1.04 %). No resistance to the remaining antibiotics was detected amongst all isolates. No differences with regard to MIC values were observed between isolates from invasive and non-invasive infections (p-value >0.05 for all antibiotics).

**Conclusion:**

GAS remains susceptible to routine-antimicrobial agents used in our low-resourced setting. Eight percent of the GAS isolates were resistant to tetracycline, and we did not observe macrolide resistance as reported in high income countries. This is the first study to report on the antimicrobial patterns of GAS in South Africa. These results address a critical gap in the available data on GAS in Africa and specifically South Africa and, thus, aid in avoiding therapeutic failures.

## Highlights

• Evidence supports the use of Penicillin remaining the treatment of choice for infections caused by *Streptococcus pyogenes.*
• Macrolide resistance rates exhibit significant variations worldwide.
• Low rates of resistance to macrolide and tetracycline were observed in Cape Town, a low-resourced country setting.
• There is no evidence supporting an association between the nature of GAS infection and antimicrobial resistance.

## 1 Introduction

*Streptococcus pyogenes* or Group A Streptococcus (GAS) is a ubiquitous Gram-positive organism. GAS can cause a range of uncomplicated as well as acute life-threatening infections, including pharyngitis, glomerulonephritis, acute rheumatic fever (ARF) and rheumatic heat disease (RHD). Infecting more than 18.1 million people and causing 500,000 deaths annually, GAS is one of the leading causes of infectious disease-related deaths worldwide [1]. Infections due to GAS are prevalent in low-income countries (up to 60 % estimates), where poverty and limited access to medical care are common [2,3]. The estimated annual incidence rate of symptomatic GAS pharyngitis is 0.4 cases per person-year, affecting over 423 million children in developing countries [1].

Appropriate antibiotic therapy of GAS pharyngitis is essential for the prevention of serious complications with the preferred treatment for confirmed GAS pharyngitis being either penicillin or amoxicillin [4]. In South Africa, penicillin V potassium (penicillin VK) or benzathine penicillin is the primary treatment of bacterial pharyngotonsillitis in both adults and children, with amoxicillin, azithromycin or clarithromycin recommended as alternatives in cases of penicillin allergy or penicillin not being available [5]. The advantages of amoxicillin therapy include increased bioavailability, improved gastrointestinal absorption and stability due to the presence of an hydroxyl (OH) radical. In situations of penicillin allergy, second-line antibiotics such as macrolides (e.g., azithromycin and erythromycin), tetracyclines and lincosamides (e.g., clindamycin) are considered effective alternate treatment options [4]. Fluctuating trends in the antibiotic resistance patterns of this organism have been reported worldwide. Treatment with macrolides and lincosamides have become more common in several paediatric settings for respiratory and skin and soft tissue infections (SSTI) and may contribute to reported increasing levels of resistance [6]. This increase in second-line antimicrobial resistance in GAS has led the Centers for Disease Control and Prevention (CDC) to identify macrolide-resistant GAS as a possible emerging threat.

Antimicrobial drugs against GAS are a vital component of RHD management, utilized to prevent suppurative complications, acute rheumatic fever (ARF), transmission rates and reduce signs and symptoms associated with GAS infections; estimates indicate a 70 % reduction in the risk of ARF through primary prevention [6]. Success was reported in Cuba in the prevention and control of ARF and RHD through programmes incorporating primary prevention with effective use of antibiotics [7]. Incorrectly-prescribed antibiotics serve to contribute to the development of antibiotic resistance [8]. Appropriate antibiotic prescription is dependent on accurate diagnosis of GAS infection with numerous attempts having been made to develop clinical prediction rules to assist physicians in distinguishing between viral and bacterial infections in the absence of microbiological tests. Behaviours resulting in inappropriate antibiotic usage stem from complex interactions among individuals, communities, and healthcare systems, shaped by epidemiological trends and influenced by cultural, political and socio-economic factors [9]. Low-income countries not only contend with a greater prevalence of infectious diseases, but also confront challenges such as a shortage of qualified healthcare professionals, unregulated antibiotic usage, widespread self-medication, limited access to unbiased information about antibiotics, inadequate availability to high-quality medicines, and limited access to newer therapeutic agents [10,11].

Resistance patterns of GAS in South Africa has never been evaluated. The present study aimed to determine the antibiotic susceptibility profiles of GAS isolated between 2016 and 2019 from invasive and non-invasive infections from hospitals in Cape Town, South Africa.

## 2 Materials and methods

### 2.1 Study design and setting

The samples used in this study were obtained from the AFRO*Strep* Registry, a continental endeavour which seeks to document *Streptococcus* pyogenes infection in Africa and collect isolates for the first biorepository of its kind [12]. Isolates comprised invasive GAS (iGAS) (n = 40/95) and non-invasive GAS (non-iGAS) (n = 55/95), from patients attending Groote Schuur hospital and community clinics in Cape Town, South Africa. Isolates were selected based on the most prevalent *emm* types described in invasive and non-invasive GAS infection in Cape Town and included nineteen different *emm* types comprising six *emm* clusters (D4, D5, E2, E3, E4, E6) (Fig. S1).

iGAS, defined as GAS isolated from sterile sources, was isolated from blood (n = 12), deep tissue (n = 12), aspirate (n = 11), and pus swabs (n = 5) from biopsy samples following surgery (Septic Arthritis, Chronic osteitis and abdominal wall abscess). Non-iGAS comprised isolates obtained from nonsterile sites, such as throat swabs (n = 10), pus swabs (n = 38), abscess (n = 2), aspirate (n = 2), pus aspirate (n = 1), percutaneous fluid (n = 1), and one isolate from an unknown source (clinical data not provided on clinical forms).

### 2.2 *Emm*-typing

The *emm*-typing procedure was performed according to previously published protocols [[13], [14], [15]]. The PCR primers were synthesized at the Department of Molecular and Cell Biology, University of Cape Town, South Africa. The PCR products were sequenced at the University of Stellenbosch Central DNA Sequencing Facility. The generated sequences were submitted to the CDC *Streptococcus pyogenes* database [16], and the respective *emm* types assigned to the sequences.

### 2.3 Antibiotic susceptibility testing

GAS isolates were previously identified by Gram stain, catalase, and serogrouping. Isolates were revived from storage at −80 °C on sheep blood agar plates [National health laboratory services (NHLS)], incubated overnight at 34–35 °C, in a microaerophilic environment, and then passaged a second time. Briefly, 3–5 colonies were mixed into Vitek 2 (BioMerieux) saline solution to a density of 0.5 McFarland using a densitometer. One hundred microliters of the suspension were combined with supplied Mueller Hinton Broth (MHB) with lysed horse blood (ThermoFisher Scientific), before adding 100 μl to each well of the supplied Sensititre plate (ThermoFisher Scientific). The plate was sealed and incubated at 34–35 °C in a microaerophilic incubator for 22 h. The plates were interpreted using the sensititre manual view box. Quality-control testing was included in each batch using *Streptococcus pneumoniae* ATCC 49619. Minimum inhibitory concentrations (MICs) were determined using Clinical Laboratory Standards Institute (CLSI) M100‐Ed31 2021 break points [17].

The antibiogram of 20 antibiotics belonging to 10 different classes including β-lactams (penicillin, amoxicillin/clavulanic acid, cefuroxime, cefotaxime, ceftriaxone, cefepime, meropenem, ertapenem), macrolides (erythromycin, azithromycin), oxazolidinones (linezolid), lincosamides (clindamycin), fluoroquinolones (levofloxacin, moxifloxacin), phenicols (chloramphenicol), lipopeptide (daptomycin), glycopeptide (vancomycin), cyclines (tetracycline, tigecycline), sulfonamides (sulfamethoxazole/trimethoprim) was performed using the Sensititre® STP6F system (ThermoFisher Scientific) following the guidelines of the manufacturer.

### 2.4 Statistical analysis

Stata V14.1 (StataCorp, College Station, TX, USA) was used to analyse the antimicrobial susceptibility test results. Descriptive statistics were expressed as numbers and percentages. Statistical differences were evaluated using Chi-squared and Fisher's exact test. A P-value of ≤0.05 was considered significant.

### 2.5 Ethical considerations

This study was approved by the University of Cape Town Human Research Ethics Committee (HREC: 826/2019; R006/2015).

## 3 Results

### 3.1 Demographic information

The GAS isolates were obtained from 22 children (age range 2–17 y/o) and 73 adult participants (18–80 y/o), with a median age of 34 years (2–80 years); 67 % male (Table S1).

### 3.2 Antimicrobial susceptibility testing to beta-lactam antibiotics

Table 1 indicates the antimicrobial resistance pattern of the 95 GAS isolates by the Sensititre® STP6F platform (20 antibiotics tested). The beta-lactam antibiotics exhibited low MIC_90_ values ranging from 0.03 to 2 μg/mL. Penicillin remained effective, with an MIC_90_ value of 0.3 μg/mL. All isolates were susceptible to amoxicillin/clavulanic acid, cefuroxime, cefotaxime, ceftriaxone, cefepime, meropenem and ertapenem (Table S2).

**Table 1:** Antimicrobial resistance pattern of the 95 GAS isolates by the Sensititre® STP6F system.

Class of antibiotic	Antibiotic	R (%)	I (%)	S (%)	MIC_90_ (μg/mL)	MIC Range (μg/mL)
β-lactams	Penicillin	0	0	100	≤0.03	0.03–4
	Amoxicillin/clavulanic acid (2:1 ratio)	0	0	100	≤2/1	2/1–16/8
	Cefuroxime	0	0	100	≤0.5	0.5–4
	Cefotaxime	0	0	100	≤0.12	0.12–4
	Ceftriaxone	0	0	100	≤0.12	0.12–2
	Cefepime	0	0	100	≤0.5	0.5–8
	Meropenem	0	0	100	≤0.25	0.25–2
	Ertapenem	0	0	100	≤0.5	0.5–4
Macrolides	Erythromycin	1	0	99	≤0.25	0.25–2
	Azithromycin	1	0	99	≤0.25	0.25–2
Oxazolidinones	Linezolid	0	0	100	1	0.25–4
Lincomycin	Clindamycin	0	0	100	≤0.12	0.12–1
Phenicol	Chloramphenicol	0	0	100	4	1–32
Lipopeptide	Daptomycin	0	0	100	0.12	0.06–2
Glycopeptides	Vancomycin	0	0	100	1	0.5–4
Fluoroquinolones	Levofloxacin	0	1	99	1	0.5–4
	Moxifloxacin	0	0	100	≤1	1–8
Tetracyclines	Tetracycline	8.4	0	91.6	1	1–8
	Tigecycline	0	0	100	0.03	0.015-0.12
Sulfonamide	Sulfamethoxazole/Trimethoprim	0	0	100	≤0.5/9.5	0.5/9.5-4/76

MIC = Minimal Inhibitory Concentration, R = resistant, I = intermediate, U = undefined, S = susceptible. Susceptibility rates have been interpreted according to CLSI breakpoints.

### 3.3 Susceptibility to macrolides, lincosamins and cyclines

Erythromycin and azithromycin indicated only 1 % exhibiting resistance across all isolates. The minimum inhibitory concentration at which 90 % of the isolates were inhibited (MIC_90_) for erythromycin and azithromycin were both 0.25 μg/mL (Table 1). Erythromycin resistance was identified in emm 43 (ST721) (Table 2). Clindamycin was susceptible in all isolates, with an MIC_90_ value of ≤0.12 μg/mL. Tetracycline was susceptible in 91.6 % of isolates and resistant in 8.4 % of isolates, with an MIC_90_ value of 1 μg/mL. Tetracycline resistance was identified in emm 76 (ST378), in both invasive and non-invasive GAS isolates, which were isolated from pus swabs (n = 3), blood (n = 2), deep tissue (n = 2) and percutaneous fluid (n = 1) (p-value >0.05) (Table S3).

**Table 2:** Profile of resistant GAS isolates in Cape Town.

Isolate number	Antibiotic resistance	Age	Gender	ST	Emm Type	Emm Cluster	GAS Infection	Site
11	EM, AZI	44	M	721	43	D4	iGAS	Blood
29	TET	30	F	378	76	E2	iGAS	Deep tissue
30	TET	40	M	378	76	E2	Non-iGAS	Pus swab
31	TET	57	F	378	76	E2	Non-iGAS	Percutaneous Fluid
32	TET	28	M	378	76	E2	Non-iGAS	Pus swab
34	TET	65	M	378	76	E2	iGAS	Deep tissue
35	TET	33	M	378	76	E2	iGAS	Blood
36	TET	20	M	378	76	E2	Non-iGAS	Pus swab
76	TET	39	M	378	76	E2	iGAS	Blood

EM, erythromycin; AZI, azithromycin; TET, tetracycline.

M, Male; F, Female.

iGAS, invasive GAS; non-iGAS, non-invasive GAS.

### 3.4 Susceptibility to other antibiotics

Ninety-nine percent of the isolates were susceptible to levofloxacin, while 1 % showed intermediate resistance. Moxifloxacin was susceptible in all isolates, with an MIC_90_ value of ≤1 μg/mL. GAS isolates showed complete susceptibility to linezolid, chloramphenicol, daptomycin and vancomycin. Interestingly, all these antibiotics showed low MIC_90_ values. Tigecycline and sulfamethoxazole/trimethoprim were susceptible in all isolates (Table S2).

## 4 Discussion

This study, which sought to determine the antimicrobial patterns among GAS isolates from invasive and non-invasive infections reports that GAS isolates from Cape Town, South Africa indicated susceptibility to the majority of routinely administered antibiotics for GAS treatment, with some resistance to tetracycline (8.33 %), azithromycin (1.04 %), and erythromycin (1.04 %) described.

Penicillin is the primary empiric choice of treatment for GAS infection. In our study, all 95 isolates were susceptible to the beta-lactam antibiotics tested. Penicillin was uniformly susceptible at low concentrations, reinforcing the fact that penicillin resistance has not been acquired in this organism in Cape Town, South Africa. The same high activity of penicillin has been reported in other countries, such as Senegal [18], Morocco [19], Germany [20], and France [21]. Interestingly, failures in penicillin treatment have been observed in GAS tonsillopharyngitis patients, which suggests the possible emergence of penicillin-resistant strains, possibly due to the production of beta-lactamases or co-pathogen interference and alteration of the upper respiratory microbiome [22]. In addition to penicillin, we found that amoxicillin was also fully active against our GAS isolates. Our results are concordant with other studies reported in Nepal [23] and Central, Eastern and Baltic European countries [24].

Macrolides such as erythromycin, are recommended as alternative therapy for patients who are allergic to penicillin. In the present study, erythromycin-GAS resistance was only detected in one isolate (1 %), which was similar to a previous Indian study which also reported 1 % resistance (n = 405) [25]. The prevalence of erythromycin resistance varies across regions, with Asia showing notably higher rates compared to Europe and the USA. High macrolide resistance has been reported in China, Pakistan, India, Korea and Japan, while Turkey, Lebanon and Taiwan, have reported low levels of resistance [[26], [27], [28]]. However, resistance to erythromycin was high in countries such as; Italy [29], Canada [30], France [31] and Portugal [32].

In this study, azithromycin resistance was observed in one isolate. A similar result was reported in Senegal using the standard disk diffusion method [18]. The rise in macrolide resistance may be attributable to the practice of wide-spread prescriptions of azithromycin for respiratory and sexually transmitted infections [33].

In this study, tetracycline resistance was identified in 8.3 % of GAS isolates, correlating with the low tetracycline resistance (6.6 %) reported in Nepal [23]. However, this was not observed in a study conducted in Serbia [34]. Tetracycline resistance has been reported in various parts of the world [35,36], even though it is not used for the treatment of GAS infections. A probable explanation could be the selective pressure from the exhaustive utilization of tetracycline in human and veterinary infections, as well as its intensive use in animal diets, with the transfer of resistance genes from animals to humans [37].

Notably, even though no resistance to moxifloxacin was observed in this study, 1 % of the isolates exhibited intermediate susceptibility to levofloxacin. This concurs with high levofloxacin susceptibility rates reported in Central, Eastern, and Baltic European countries [24].

Several factors need to be considered when selecting an antibiotic for treatment. Each isolated strain should ideally be evaluated for antibiotic susceptibility for appropriate, targeted therapy. The antibiotic treatment should be coupled with suitable preventative approaches that should include educating the general population on hygiene and discouraging habitual self-medication practices, as well as improved consultation between clinical microbiologists and clinicians to improve public health.

In conclusion, GAS isolates from Cape Town, South Africa remained susceptible to the majority of routine antimicrobials used for GAS treatment. While antibiotic resistance was not detected in these isolates, early diagnosis and appropriate antibiotic treatment are necessary to treat acute pharyngitis and prevent subsequent infections, such as ARF and RHD. Eight percent of GAS isolates studied were resistant to tetracycline, while all isolates remain sensitive to penicillin. Antimicrobial susceptibility profiles of invasive and non-invasive GAS infection showed no significant differences. Knowledge of the prevalence of resistance and MIC trends of commonly used antibiotics for *Streptococcus pyogenes* will aid in avoiding therapeutic failures, limiting the acquisition of resistance, and informing future vaccine development. Future work will include genomic characterisation of these isolates to describe their relatedness and correlate the phenotypic results obtained with the presence of resistance elements.

## Funding

Research reported in this publication was supported by the 10.13039/501100001322South African Medical Research Council (10.13039/501100001322SAMRC) under a Self-Initiated Research Grant. K.R., K.E. and T.S. are also funded by a grant from the 10.13039/501100001322SAMRC. The views and opinions expressed are those of the authors and do not necessarily represent the official views of the SAMRC.

## CRediT authorship contribution statement

**Kimona Rampersadh:** Writing – review & editing, Writing – original draft, Visualization, Validation, Project administration, Methodology, Investigation, Formal analysis, Data curation, Conceptualization. **Kelin C. Engel:** Writing – review & editing, Project administration, Methodology, Investigation. **Mark E. Engel:** Writing – review & editing, Validation, Supervision, Methodology, Funding acquisition, Conceptualization. **Clinton Moodley:** Writing – review & editing, Visualization, Validation, Methodology, Investigation, Formal analysis, Data curation, Conceptualization.

## Declaration of competing interest

The authors declare that they have no known competing financial interests or personal relationships that could have appeared to influence the work reported in this paper.
