# Supplementary material for: A survey of antibiotic resistance patterns among Group A Streptococcus isolated from invasive and non-invasive infections in Cape Town, South Africa
Source: Heliyon. 2024 Jun 26;10(13):e33694. doi: 10.1016/j.heliyon.2024.e33694 (PMC11261099; doi:10.1016/j.heliyon.2024.e33694)
Supplement: Multimedia component 1 [file mmc1.docx]

**Appendix**

**Figure S1: Emm types of the invasive and non-invasive GAS isolates.**

**Table S1. Characteristics of the 95 GAS isolates.**

| **ID** | **Age** | **Gender** | **ET** | **Invasive** | **Site** |
| --- | --- | --- | --- | --- | --- |
| 1 | 44 | M | 25.1 | Yes | Deep tissue |
| 2 | 40 | M | 25.1 | Yes | Pus swab |
| 3 | 19 | F | 25.1 | No | Pus swab |
| 4 | 23 | F | 25.1 | No | Pus swab |
| 5 | 63 | M | 33 | No | Pus swab |
| 6 | 28 | M | 33 | No | Pus swab |
| 7 | 27 | M | 33 | Yes | Pus swab |
| 8 | 64 | F | 33 | Yes | Aspirate |
| 9 | 59 | M | 43.7 | Yes | Blood |
| 10 | 16 | M | 43.7 | No | Pus swab |
| 11 | 44 | M | 43.7 | Yes | Blood |
| 12 | 11 | M | 43.7 | No | Pus swab |
| 13 | 35 | M | 44 | Yes | Blood |
| 14 | 47 | M | 44 | No | Pus swab |
| 15 | 42 | F | 44 | Yes | Deep tissue |
| 16 | 31 | F | 44 | No | Pus swab |
| 17 | 63 | M | 49 | No | Abscess |
| 18 | 57 | F | 49 | Yes | Aspirate |
| 19 | 46 | F | 49 | Yes | Aspirate |
| 20 | 52 | M | 49 | No | Pus swab |
| 21 | 61 | M | 53 | Yes | Aspirate |
| 22 | 3 | F | 53 | No | Pus swab |
| 23 | 40 | M | 53 | No | Pus swab |
| 24 | 43 | M | 53 | Yes | Blood |
| 25 | 28 | M | 65.4 | No | Pus swab |
| 26 | 48 | F | 65.4 | Yes | Aspirate |
| 27 | 50 | M | 65.4 | No | Pus swab |
| 28 | 46 | F | 65.4 | Yes | Deep tissue |
| 29 | 30 | F | 76 | Yes | Deep tissue |
| 30 | 40 | M | 76 | No | Pus swab |
| 31 | 57 | F | 76 | No | percutaneous fluid |
| 32 | 28 | M | 76 | No | Pus swab |
| 33 | 28 | M | 81 | Yes | Blood |
| 34 | 65 | M | 76 | Yes | Deep tissue |
| 35 | 33 | M | 76 | Yes | Blood |
| 36 | 20 | M | 76 | No | Pus swab |
| 37 | 40 | M | 80 | Yes | Blood |
| 38 | 42 | M | 80 | No | Pus swab |
| 39 | 61 | F | 44 | Yes | Aspirate |
| 40 | 9 | M | 80 | No | Abscess |
| 41 | 17 | M | 80 | Yes | Pus swab |
| 42 | 27 | M | 80 | Yes | Blood |
| 43 | 22 | F | 80 | No | Pus swab |
| 44 | 41 | M | 64.4 | No | Pus swab |
| 45 | 19 | M | 81 | No | NS |
| 46 | 78 | M | 81 | Yes | Aspirate |
| 47 | 26 | M | 81 | Yes | Deep tissue |
| 48 | 26 | F | 81 | No | Pus swab |
| 49 | 4 | M | 81 | Yes | Pus swab |
| 50 | 20 | M | 81 | No | Pus swab |
| 51 | 59 | M | 81 | Yes | Blood |
| 52 | 21 | M | 81 | No | pus aspirate |
| 53 | 2 | M | 82 | No | Pus swab |
| 54 | 66 | M | 82 | Yes | Aspirate |
| 55 | 29 | F | 87 | Yes | Deep tissue |
| 56 | 70 | F | 87 | No | Pus swab |
| 57 | 7 | M | 87 | No | Pus swab |
| 58 | 8 | M | 87 | Yes | Aspirate |
| 59 | 4 | M | 177 | No | Pus swab |
| 60 | 52 | M | 89 | Yes | Deep tissue |
| 61 | 21 | F | 89 | No | Pus swab |
| 62 | 50 | F | 92 | No | Pus swab |
| 63 | 22 | M | 92 | Yes | Deep tissue |
| 64 | 35 | M | 92 | Yes | Pus swab |
| 65 | 20 | M | 92 | No | Aspirate |
| 66 | 61 | F | 92 | Yes | Blood |
| 67 | 31 | F | 92 | No | Pus swab |
| 68 | 27 | M | 92 | No | Pus swab |
| 69 | 19 | M | 92 | No | Pus swab |
| 70 | 36 | M | 92 | Yes | Deep tissue |
| 71 | 55 | M | 92 | Yes | Blood |
| 72 | 55 | M | 92 | No | Pus swab |
| 73 | 39 | F | 92 | No | Pus swab |
| 74 | 55 | M | 92 | No | Aspirate |
| 75 | 52 | M | 92 | No | Pus swab |
| 76 | 39 | M | 76 | Yes | Blood |
| 77 | 43 | M | 75 | No | Pus swab |
| 78 | 16 | F | 183.2 | Yes | Aspirate |
| 79 | 29 | M | 183.2 | No | Pus swab |
| 80 | 30 | F | 183.2 | Yes | Deep tissue |
| 81 | 30 | F | 183.2 | No | Pus swab |
| 82 | 9 | M | 184 | No | Pus swab |
| 83 | 79 | F | 184 | Yes | Deep tissue |
| 84 | 19 | M | 184 | No | Pus swab |
| 85 | 80 | M | 184 | Yes | Aspirate |
| 86 | 9 | M | 49 | No | Throat swab |
| 87 | 13 | F | 49 | No | Throat swab |
| 88 | 12 | F | 44 | No | Throat swab |
| 89 | 8 | M | 44 | No | Throat swab |
| 90 | 13 | M | 80 | No | Throat swab |
| 91 | 7 | F | 80 | No | Throat swab |
| 92 | 12 | F | 87 | No | Throat swab |
| 93 | 7 | M | 87 | No | Throat swab |
| 94 | 7 | F | 82 | No | Throat swab |
| 95 | 7 | F | 82 | No | Throat swab |

M = male, F = female.

| **Antibiotic** | **iGAS**  **(n=40)** | **Non-iGAS**  **(n=55)** | **P-value (Fisher's Exact)** |
| --- | --- | --- | --- |
| Penicillin | 0 | 0 | . |
| Amoxicillin/clavulanic acid | 0 | 0 | . |
| Cefuroxime | 0 | 0 | . |
| Cefotaxime | 0 | 0 | . |
| Ceftriaxone | 0 | 0 | . |
| Cefepime | 0 | 0 | . |
| Meropenem | 0 | 0 | . |
| Ertapenem | 0 | 0 | . |
| Erythromycin | 1 | 0 | 0,42 |
| Azithromycin | 1 | 0 | 0,42 |
| Linezolid | 0 | 0 | . |
| Clindamycin | 0 | 0 | . |
| Levofloxacin**^*^** | 1 | 0 | 0,42 |
| Moxifloxacin | 0 | 0 | . |
| Chloramphenicol | 0 | 0 | . |
| Daptomycin | 0 | 0 | . |
| Vancomycin | 0 | 0 | . |
| Tetracycline | 4 | 4 | 0,45 |
| Tigecycline | 0 | 0 | . |
| Sulfamethoxazole/ Trimethoprim | 0 | 0 | . |
| iGAS, invasive GAS; non-iGAS, non-invasive GAS  * , one isolate with intermediate resistance to levofloxacin.  . , unable to calculate p-value | | | |

**Table S2. Antibiotic resistance profile according to clinical syndrome.**

**Table S3. MICs of the antibiotics tested with the Sensititre STP platform.**

| ID | ET | | iGAS | Site | PEN | AUG2 | FUR | FOT | AXO | FEP | MERO | ETP | ERY | AZI | LZD | CLI | LEVO | MXF | CHL | DAP | VAN | TET | TGC | SXT |
| --- | --- | --- | --- | --- | --- | --- | --- | --- | --- | --- | --- | --- | --- | --- | --- | --- | --- | --- | --- | --- | --- | --- | --- | --- |
| 1 | 25.1 | | Yes | DT | ≤0.03 | ≤2/1 | ≤0.5 | ≤0.12 | ≤0.12 | ≤0.5 | ≤0.25 | ≤0.5 | ≤0.25 | ≤0.25 | 1 | ≤0.12 | 1 | ≤1 | 4 | ≤0.06 | ≤0.5 | ≤1 | 0.03 | ≤0.5/9.5 |
| 2 | 25.1 | Yes | | PS | ≤0.03 | ≤2/1 | ≤0.5 | ≤0.12 | ≤0.12 | ≤0.5 | ≤0.25 | ≤0.5 | ≤0.25 | ≤0.25 | 1 | ≤0.12 | 1 | ≤1 | 4 | ≤0.06 | ≤0.5 | ≤1 | 0.03 | ≤0.5/9.5 |
| 3 | 25.1 | | No | PS | ≤0.03 | ≤2/1 | ≤0.5 | ≤0.12 | ≤0.12 | ≤0.5 | ≤0.25 | ≤0.5 | ≤0.25 | ≤0.25 | 1 | ≤0.12 | 1 | ≤1 | 4 | ≤0.06 | ≤0.5 | ≤1 | 0.03 | ≤0.5/9.5 |
| 4 | 25.1 | | No | PS | ≤0.03 | ≤2/1 | ≤0.5 | ≤0.12 | ≤0.12 | ≤0.5 | ≤0.25 | ≤0.5 | ≤0.25 | ≤0.25 | 1 | ≤0.12 | 1 | ≤1 | 4 | 0.12 | ≤0.5 | ≤1 | 0.03 | ≤0.5/9.5 |
| 5 | 33 | | No | PS | ≤0.03 | ≤2/1 | ≤0.5 | ≤0.12 | ≤0.12 | ≤0.5 | ≤0.25 | ≤0.5 | ≤0.25 | ≤0.25 | 1 | ≤0.12 | 1 | ≤1 | 4 | 0.12 | ≤0.5 | ≤1 | 0.03 | ≤0.5/9.5 |
| 6 | 33 | | No | PS | ≤0.03 | ≤2/1 | ≤0.5 | ≤0.12 | ≤0.12 | ≤0.5 | ≤0.25 | ≤0.5 | ≤0.25 | ≤0.25 | 1 | ≤0.12 | 1 | ≤1 | 4 | 0.5 | 1 | ≤1 | 0.03 | ≤0.5/9.5 |
| 7 | 33 | | Yes | PS | ≤0.03 | ≤2/1 | ≤0.5 | ≤0.12 | ≤0.12 | ≤0.5 | ≤0.25 | ≤0.5 | ≤0.25 | ≤0.25 | 1 | ≤0.12 | 1 | ≤1 | 4 | 0.25 | 1 | ≤1 | 0.03 | ≤0.5/9.5 |
| 8 | 33 | | Yes | ASP | ≤0.03 | ≤2/1 | ≤0.5 | ≤0.12 | ≤0.12 | ≤0.5 | ≤0.25 | ≤0.5 | ≤0.25 | ≤0.25 | 1 | ≤0.12 | 1 | ≤1 | 4 | 0.12 | 1 | ≤1 | 0.03 | ≤0.5/9.5 |
| 9 | 43 | | Yes | B | ≤0.03 | ≤2/1 | ≤0.5 | ≤0.12 | ≤0.12 | ≤0.5 | ≤0.25 | ≤0.5 | ≤0.25 | ≤0.25 | 1 | ≤0.12 | 1 | ≤1 | 4 | ≤0.06 | ≤0.5 | ≤1 | 0.03 | ≤0.5/9.5 |
| 10 | 43 | | No | PS | ≤0.03 | ≤2/1 | ≤0.5 | ≤0.12 | ≤0.12 | ≤0.5 | ≤0.25 | ≤0.5 | ≤0.25 | ≤0.25 | 1 | ≤0.12 | ≤0.5 | ≤1 | 4 | ≤0.06 | 1 | ≤1 | 0.03 | ≤0.5/9.5 |
| 11 | 43 | | Yes | B | ≤0.03 | ≤2/1 | ≤0.5 | ≤0.12 | ≤0.12 | ≤0.5 | ≤0.25 | ≤0.5 | >2 | >2 | 1 | ≤0.12 | 1 | ≤1 | 4 | ≤0.06 | 1 | ≤1 | 0.03 | ≤0.5/9.5 |
| 12 | 43 | | No | PS | ≤0.03 | ≤2/1 | ≤0.5 | ≤0.12 | ≤0.12 | ≤0.5 | ≤0.25 | ≤0.5 | ≤0.25 | ≤0.25 | 1 | ≤0.12 | 1 | ≤1 | 4 | ≤0.06 | ≤0.5 | ≤1 | 0.03 | ≤0.5/9.5 |
| 13 | 44 | | Yes | B | ≤0.03 | ≤2/1 | ≤0.5 | ≤0.12 | ≤0.12 | ≤0.5 | ≤0.25 | ≤0.5 | ≤0.25 | ≤0.25 | 2 | ≤0.12 | 1 | ≤1 | 4 | ≤0.06 | 1 | ≤1 | 0.03 | ≤0.5/9.5 |
| 14 | 44 | | No | PS | ≤0.03 | ≤2/1 | ≤0.5 | ≤0.12 | ≤0.12 | ≤0.5 | ≤0.25 | ≤0.5 | ≤0.25 | ≤0.25 | 1 | ≤0.12 | 1 | ≤1 | 4 | ≤0.06 | ≤0.5 | ≤1 | 0.03 | ≤0.5/9.5 |
| 15 | 44 | | Yes | DT | ≤0.03 | ≤2/1 | ≤0.5 | ≤0.12 | ≤0.12 | ≤0.5 | ≤0.25 | ≤0.5 | ≤0.25 | ≤0.25 | 1 | ≤0.12 | 1 | ≤1 | 4 | ≤0.06 | ≤0.5 | ≤1 | 0.03 | ≤0.5/9.5 |
| 16 | 44 | | No | PS | ≤0.03 | ≤2/1 | ≤0.5 | ≤0.12 | ≤0.12 | ≤0.5 | ≤0.25 | ≤0.5 | ≤0.25 | ≤0.25 | 1 | ≤0.12 | 1 | ≤1 | 4 | 0.12 | ≤0.5 | ≤1 | 0.03 | ≤0.5/9.5 |
| 17 | 49 | | No | AB | ≤0.03 | ≤2/1 | ≤0.5 | ≤0.12 | ≤0.12 | ≤0.5 | ≤0.25 | ≤0.5 | ≤0.25 | ≤0.25 | 1 | ≤0.12 | 1 | ≤1 | 2 | ≤0.06 | ≤0.5 | ≤1 | 0.03 | 1/19 |
| 18 | 49 | | Yes | ASP | ≤0.03 | ≤2/1 | ≤0.5 | ≤0.12 | ≤0.12 | ≤0.5 | ≤0.25 | ≤0.5 | ≤0.25 | ≤0.25 | 1 | ≤0.12 | 1 | ≤1 | 4 | ≤0.06 | ≤0.5 | ≤1 | 0.03 | ≤0.5/9.5 |
| 19 | 49 | | Yes | ASP | ≤0.03 | ≤2/1 | ≤0.5 | ≤0.12 | ≤0.12 | ≤0.5 | ≤0.25 | ≤0.5 | ≤0.25 | ≤0.25 | 1 | ≤0.12 | 1 | ≤1 | 4 | ≤0.06 | ≤0.5 | ≤1 | 0.03 | ≤0.5/9.5 |
| 20 | 49 | | No | PS | ≤0.03 | ≤2/1 | ≤0.5 | ≤0.12 | ≤0.12 | ≤0.5 | ≤0.25 | ≤0.5 | ≤0.25 | ≤0.25 | 1 | ≤0.12 | 1 | ≤1 | 2 | ≤0.06 | ≤0.5 | ≤1 | 0.03 | ≤0.5/9.5 |
| 21 | 53 | | Yes | ASP | ≤0.03 | ≤2/1 | ≤0.5 | ≤0.12 | ≤0.12 | ≤0.5 | ≤0.25 | ≤0.5 | ≤0.25 | ≤0.25 | 1 | ≤0.12 | 1 | ≤1 | 4 | ≤0.06 | ≤0.5 | ≤1 | 0.03 | ≤0.5/9.5 |
| 22 | 53 | | No | PS | ≤0.03 | ≤2/1 | ≤0.5 | ≤0.12 | ≤0.12 | ≤0.5 | ≤0.25 | ≤0.5 | ≤0.25 | ≤0.25 | 1 | ≤0.12 | 1 | ≤1 | 4 | ≤0.06 | ≤0.5 | ≤1 | 0.03 | ≤0.5/9.5 |
| 23 | 53 | | No | PS | ≤0.03 | ≤2/1 | ≤0.5 | ≤0.12 | ≤0.12 | ≤0.5 | ≤0.25 | ≤0.5 | ≤0.25 | ≤0.25 | 1 | ≤0.12 | 1 | ≤1 | 4 | ≤0.06 | ≤0.5 | ≤1 | 0.03 | ≤0.5/9.5 |
| 24 | 53 | | Yes | B | ≤0.03 | ≤2/1 | ≤0.5 | ≤0.12 | ≤0.12 | ≤0.5 | ≤0.25 | ≤0.5 | ≤0.25 | ≤0.25 | 1 | ≤0.12 | 1 | ≤1 | 4 | ≤0.06 | ≤0.5 | ≤1 | 0.03 | ≤0.5/9.5 |
| 25 | 65.4 | | No | PS | ≤0.03 | ≤2/1 | ≤0.5 | ≤0.12 | ≤0.12 | ≤0.5 | ≤0.25 | ≤0.5 | ≤0.25 | ≤0.25 | 1 | ≤0.12 | 1 | ≤1 | 4 | ≤0.06 | ≤0.5 | ≤1 | 0.03 | ≤0.5/9.5 |
| 26 | 65.4 | | Yes | ASP | ≤0.03 | ≤2/1 | ≤0.5 | ≤0.12 | ≤0.12 | ≤0.5 | ≤0.25 | ≤0.5 | ≤0.25 | ≤0.25 | 1 | ≤0.12 | 1 | ≤1 | 4 | ≤0.06 | ≤0.5 | ≤1 | 0.03 | ≤0.5/9.5 |
| 27 | 65.4 | | No | PS | ≤0.03 | ≤2/1 | ≤0.5 | ≤0.12 | ≤0.12 | ≤0.5 | ≤0.25 | ≤0.5 | ≤0.25 | ≤0.25 | 1 | ≤0.12 | 1 | ≤1 | 4 | 0.12 | ≤0.5 | ≤1 | 0.03 | ≤0.5/9.5 |
| 28 | 65.4 | | Yes | DT | ≤0.03 | ≤2/1 | ≤0.5 | ≤0.12 | ≤0.12 | ≤0.5 | ≤0.25 | ≤0.5 | ≤0.25 | ≤0.25 | 1 | ≤0.12 | 2 | ≤1 | 4 | ≤0.06 | ≤0.5 | ≤1 | 0.03 | ≤0.5/9.5 |
| 29 | 76 | | Yes | DT | 0.06 | ≤2/1 | ≤0.5 | ≤0.12 | ≤0.12 | ≤0.5 | ≤0.25 | ≤0.5 | ≤0.25 | ≤0.25 | 1 | ≤0.12 | 1 | ≤1 | 4 | ≤0.06 | 1 | >8 | 0.06 | ≤0.5/9.5 |
| 30 | 76 | | No | PS | ≤0.03 | ≤2/1 | ≤0.5 | ≤0.12 | ≤0.12 | ≤0.5 | ≤0.25 | ≤0.5 | ≤0.25 | ≤0.25 | 1 | ≤0.12 | 1 | ≤1 | 4 | ≤0.06 | ≤0.5 | >8 | 0.06 | ≤0.5/9.5 |
| 31 | 76 | | No | PF | ≤0.03 | ≤2/1 | ≤0.5 | ≤0.12 | ≤0.12 | ≤0.5 | ≤0.25 | ≤0.5 | ≤0.25 | ≤0.25 | 1 | ≤0.12 | ≤0.5 | ≤1 | 4 | ≤0.06 | 1 | >8 | 0.06 | ≤0.5/9.5 |
| 32 | 76 | | No | PS | ≤0.03 | ≤2/1 | ≤0.5 | ≤0.12 | ≤0.12 | ≤0.5 | ≤0.25 | ≤0.5 | ≤0.25 | ≤0.25 | 1 | ≤0.12 | 1 | ≤1 | 4 | ≤0.06 | ≤0.5 | >8 | 0.06 | ≤0.5/9.5 |
| 33 | 81 | | Yes | B | ≤0.03 | ≤2/1 | ≤0.5 | ≤0.12 | ≤0.12 | ≤0.5 | ≤0.25 | ≤0.5 | ≤0.25 | ≤0.25 | 1 | ≤0.12 | 1 | ≤1 | 4 | ≤0.06 | ≤0.5 | ≤1 | 0.03 | ≤0.5/9.5 |
| 34 | 76 | | Yes | DT | ≤0.03 | ≤2/1 | ≤0.5 | ≤0.12 | ≤0.12 | ≤0.5 | ≤0.25 | ≤0.5 | ≤0.25 | ≤0.25 | 1 | ≤0.12 | 4 | ≤1 | 4 | 0.12 | ≤0.5 | >8 | 0.06 | ≤0.5/9.5 |
| 35 | 76 | | Yes | B | ≤0.03 | ≤2/1 | ≤0.5 | ≤0.12 | ≤0.12 | ≤0.5 | ≤0.25 | ≤0.5 | ≤0.25 | ≤0.25 | 1 | ≤0.12 | 1 | ≤1 | 4 | 0.12 | ≤0.5 | >8 | 0.06 | ≤0.5/9.5 |
| 36 | 76 | | No | PS | ≤0.03 | ≤2/1 | ≤0.5 | ≤0.12 | ≤0.12 | ≤0.5 | ≤0.25 | ≤0.5 | ≤0.25 | ≤0.25 | 1 | ≤0.12 | 1 | ≤1 | 4 | ≤0.06 | 1 | >8 | 0.06 | ≤0.5/9.5 |
| 37 | 80 | | Yes | B | ≤0.03 | ≤2/1 | ≤0.5 | ≤0.12 | ≤0.12 | ≤0.5 | ≤0.25 | ≤0.5 | ≤0.25 | ≤0.25 | 1 | ≤0.12 | 1 | ≤1 | 4 | ≤0.06 | ≤0.5 | ≤1 | 0.03 | ≤0.5/9.5 |
| 38 | 80 | | No | PS | ≤0.03 | ≤2/1 | ≤0.5 | ≤0.12 | ≤0.12 | ≤0.5 | ≤0.25 | ≤0.5 | ≤0.25 | ≤0.25 | 1 | ≤0.12 | 1 | ≤1 | 4 | ≤0.06 | 1 | ≤1 | 0.03 | ≤0.5/9.5 |
| 39 | 44 | | Yes | ASP | ≤0.03 | ≤2/1 | ≤0.5 | ≤0.12 | ≤0.12 | ≤0.5 | ≤0.25 | ≤0.5 | ≤0.25 | ≤0.25 | 1 | ≤0.12 | 1 | ≤1 | 4 | ≤0.06 | ≤0.5 | ≤1 | ≤0.015 | ≤0.5/9.5 |
| 40 | 80 | | No | AB | ≤0.03 | ≤2/1 | ≤0.5 | ≤0.12 | ≤0.12 | ≤0.5 | ≤0.25 | ≤0.5 | ≤0.25 | ≤0.25 | 1 | ≤0.12 | 1 | ≤1 | 4 | ≤0.06 | ≤0.5 | ≤1 | 0.03 | ≤0.5/9.5 |
| 41 | 80 | | Yes | PS | ≤0.03 | ≤2/1 | ≤0.5 | ≤0.12 | ≤0.12 | ≤0.5 | ≤0.25 | ≤0.5 | ≤0.25 | ≤0.25 | 1 | ≤0.12 | 1 | ≤1 | 2 | 0.12 | ≤0.5 | ≤1 | 0.03 | ≤0.5/9.5 |
| 42 | 80 | | Yes | B | ≤0.03 | ≤2/1 | ≤0.5 | ≤0.12 | ≤0.12 | ≤0.5 | ≤0.25 | ≤0.5 | ≤0.25 | ≤0.25 | 1 | ≤0.12 | 1 | ≤1 | 4 | 0.12 | 1 | ≤1 | 0.03 | ≤0.5/9.5 |
| 43 | 80 | | No | PS | ≤0.03 | ≤2/1 | ≤0.5 | ≤0.12 | ≤0.12 | ≤0.5 | ≤0.25 | ≤0.5 | ≤0.25 | ≤0.25 | 1 | ≤0.12 | 1 | ≤1 | 4 | ≤0.06 | ≤0.5 | ≤1 | 0.03 | ≤0.5/9.5 |
| 44 | 64.4 | | No | PS | ≤0.03 | ≤2/1 | ≤0.5 | ≤0.12 | ≤0.12 | ≤0.5 | ≤0.25 | ≤0.5 | ≤0.25 | ≤0.25 | 1 | ≤0.12 | 1 | ≤1 | 4 | ≤0.06 | ≤0.5 | ≤1 | 0.03 | ≤0.5/9.5 |
| 45 | 81 | | No | NS | ≤0.03 | ≤2/1 | ≤0.5 | ≤0.12 | ≤0.12 | ≤0.5 | ≤0.25 | ≤0.5 | ≤0.25 | ≤0.25 | 1 | ≤0.12 | 1 | ≤1 | 2 | ≤0.06 | ≤0.5 | ≤1 | 0.03 | ≤0.5/9.5 |
| 46 | 81 | | Yes | ASP | ≤0.03 | ≤2/1 | ≤0.5 | ≤0.12 | ≤0.12 | ≤0.5 | ≤0.25 | ≤0.5 | ≤0.25 | ≤0.25 | 1 | ≤0.12 | 1 | ≤1 | 4 | ≤0.06 | ≤0.5 | ≤1 | 0.03 | ≤0.5/9.5 |
| 47 | 81 | | Yes | DT | ≤0.03 | ≤2/1 | ≤0.5 | ≤0.12 | ≤0.12 | ≤0.5 | ≤0.25 | ≤0.5 | ≤0.25 | ≤0.25 | 1 | ≤0.12 | 1 | ≤1 | 4 | ≤0.06 | ≤0.5 | ≤1 | 0.03 | ≤0.5/9.5 |
| 48 | 81 | | No | PS | ≤0.03 | ≤2/1 | ≤0.5 | ≤0.12 | ≤0.12 | ≤0.5 | ≤0.25 | ≤0.5 | ≤0.25 | ≤0.25 | 1 | ≤0.12 | 1 | ≤1 | 4 | ≤0.06 | ≤0.5 | ≤1 | 0.03 | ≤0.5/9.5 |
| 49 | 81 | | Yes | PS | ≤0.03 | ≤2/1 | ≤0.5 | ≤0.12 | ≤0.12 | ≤0.5 | ≤0.25 | ≤0.5 | ≤0.25 | ≤0.25 | 1 | ≤0.12 | 1 | ≤1 | 4 | ≤0.06 | ≤0.5 | ≤1 | 0.03 | ≤0.5/9.5 |
| 50 | 81 | | No | PS | ≤0.03 | ≤2/1 | ≤0.5 | ≤0.12 | ≤0.12 | ≤0.5 | ≤0.25 | ≤0.5 | ≤0.25 | ≤0.25 | 1 | ≤0.12 | 1 | ≤1 | 4 | ≤0.06 | ≤0.5 | ≤1 | 0.03 | ≤0.5/9.5 |
| 51 | 81 | | Yes | B | ≤0.03 | ≤2/1 | ≤0.5 | ≤0.12 | ≤0.12 | ≤0.5 | ≤0.25 | ≤0.5 | ≤0.25 | ≤0.25 | 1 | ≤0.12 | 1 | ≤1 | 4 | 0.25 | ≤0.5 | ≤1 | 0.03 | ≤0.5/9.5 |
| 52 | 81 | | No | PA | ≤0.03 | ≤2/1 | ≤0.5 | ≤0.12 | ≤0.12 | ≤0.5 | ≤0.25 | ≤0.5 | ≤0.25 | ≤0.25 | 1 | ≤0.12 | 1 | ≤1 | 4 | 0.25 | ≤0.5 | ≤1 | 0.03 | ≤0.5/9.5 |
| 53 | 82 | | No | PS | ≤0.03 | ≤2/1 | ≤0.5 | ≤0.12 | ≤0.12 | ≤0.5 | ≤0.25 | ≤0.5 | ≤0.25 | ≤0.25 | 1 | ≤0.12 | 1 | ≤1 | 4 | ≤0.06 | ≤0.5 | ≤1 | 0.03 | ≤0.5/9.5 |
| 54 | 82 | | Yes | ASP | ≤0.03 | ≤2/1 | ≤0.5 | ≤0.12 | ≤0.12 | ≤0.5 | ≤0.25 | ≤0.5 | ≤0.25 | ≤0.25 | 1 | ≤0.12 | 1 | ≤1 | 4 | 0.12 | ≤0.5 | ≤1 | 0.03 | ≤0.5/9.5 |
| 55 | 87 | | Yes | DT | ≤0.03 | ≤2/1 | ≤0.5 | ≤0.12 | ≤0.12 | ≤0.5 | ≤0.25 | ≤0.5 | ≤0.25 | ≤0.25 | 1 | ≤0.12 | 1 | ≤1 | 4 | ≤0.06 | ≤0.5 | ≤1 | 0.03 | ≤0.5/9.5 |
| 56 | 87 | | No | PS | ≤0.03 | ≤2/1 | ≤0.5 | ≤0.12 | ≤0.12 | ≤0.5 | ≤0.25 | ≤0.5 | ≤0.25 | ≤0.25 | 1 | ≤0.12 | 1 | ≤1 | 4 | 0.25 | ≤0.5 | ≤1 | 0.03 | ≤0.5/9.5 |
| 57 | 87 | | No | PS | ≤0.03 | ≤2/1 | ≤0.5 | ≤0.12 | ≤0.12 | ≤0.5 | ≤0.25 | ≤0.5 | ≤0.25 | ≤0.25 | 1 | ≤0.12 | 1 | ≤1 | 4 | 0.12 | ≤0.5 | ≤1 | 0.03 | ≤0.5/9.5 |
| 58 | 87 | | Yes | ASP | ≤0.03 | ≤2/1 | ≤0.5 | ≤0.12 | ≤0.12 | ≤0.5 | ≤0.25 | ≤0.5 | ≤0.25 | ≤0.25 | 1 | ≤0.12 | 1 | ≤1 | 4 | 0.12 | ≤0.5 | ≤1 | 0.03 | ≤0.5/9.5 |
| 59 | 177 | | No | PS | ≤0.03 | ≤2/1 | ≤0.5 | ≤0.12 | ≤0.12 | ≤0.5 | ≤0.25 | ≤0.5 | ≤0.25 | ≤0.25 | 1 | ≤0.12 | 1 | ≤1 | 2 | ≤0.06 | ≤0.5 | ≤1 | 0.03 | ≤0.5/9.5 |
| 60 | 89 | | Yes | DT | ≤0.03 | ≤2/1 | ≤0.5 | ≤0.12 | ≤0.12 | ≤0.5 | ≤0.25 | ≤0.5 | ≤0.25 | ≤0.25 | 1 | ≤0.12 | 1 | ≤1 | 4 | ≤0.06 | 1 | ≤1 | 0.03 | ≤0.5/9.5 |
| 61 | 89 | | No | PS | ≤0.03 | ≤2/1 | ≤0.5 | ≤0.12 | ≤0.12 | ≤0.5 | ≤0.25 | ≤0.5 | ≤0.25 | ≤0.25 | 1 | ≤0.12 | 1 | ≤1 | 4 | ≤0.06 | ≤0.5 | ≤1 | 0.03 | ≤0.5/9.5 |
| 62 | 92 | | No | PS | ≤0.03 | ≤2/1 | ≤0.5 | ≤0.12 | ≤0.12 | ≤0.5 | ≤0.25 | ≤0.5 | ≤0.25 | ≤0.25 | 1 | ≤0.12 | 1 | ≤1 | 4 | 0.25 | ≤0.5 | ≤1 | 0.03 | ≤0.5/9.5 |
| 63 | 92 | | Yes | DT | ≤0.03 | ≤2/1 | ≤0.5 | ≤0.12 | ≤0.12 | ≤0.5 | ≤0.25 | ≤0.5 | ≤0.25 | ≤0.25 | 1 | ≤0.12 | 1 | ≤1 | 4 | 0.12 | ≤0.5 | ≤1 | 0.03 | ≤0.5/9.5 |
| 64 | 92 | | Yes | PS | ≤0.03 | ≤2/1 | ≤0.5 | ≤0.12 | ≤0.12 | ≤0.5 | ≤0.25 | ≤0.5 | ≤0.25 | ≤0.25 | 1 | ≤0.12 | 1 | ≤1 | 4 | ≤0.06 | ≤0.5 | ≤1 | 0.03 | ≤0.5/9.5 |
| 65 | 92 | | No | ASP | ≤0.03 | ≤2/1 | ≤0.5 | ≤0.12 | ≤0.12 | ≤0.5 | ≤0.25 | ≤0.5 | ≤0.25 | ≤0.25 | 1 | ≤0.12 | 1 | ≤1 | 4 | ≤0.06 | ≤0.5 | ≤1 | 0.03 | ≤0.5/9.5 |
| 66 | 92 | | Yes | B | ≤0.03 | ≤2/1 | ≤0.5 | ≤0.12 | ≤0.12 | ≤0.5 | ≤0.25 | ≤0.5 | ≤0.25 | ≤0.25 | 1 | ≤0.12 | 1 | ≤1 | 4 | ≤0.06 | ≤0.5 | ≤1 | 0.03 | ≤0.5/9.5 |
| 67 | 92 | | No | PS | ≤0.03 | ≤2/1 | ≤0.5 | ≤0.12 | ≤0.12 | ≤0.5 | ≤0.25 | ≤0.5 | ≤0.25 | ≤0.25 | 1 | ≤0.12 | 1 | ≤1 | 4 | ≤0.06 | ≤0.5 | ≤1 | 0.03 | ≤0.5/9.5 |
| 68 | 92 | | No | PS | ≤0.03 | ≤2/1 | ≤0.5 | ≤0.12 | ≤0.12 | ≤0.5 | ≤0.25 | ≤0.5 | ≤0.25 | ≤0.25 | 1 | ≤0.12 | 1 | ≤1 | 4 | 0.12 | ≤0.5 | ≤1 | 0.03 | ≤0.5/9.5 |
| 69 | 92 | | No | PS | ≤0.03 | ≤2/1 | ≤0.5 | ≤0.12 | ≤0.12 | ≤0.5 | ≤0.25 | ≤0.5 | ≤0.25 | ≤0.25 | 1 | ≤0.12 | 1 | ≤1 | 4 | ≤0.06 | 1 | ≤1 | 0.03 | ≤0.5/9.5 |
| 70 | 92 | | Yes | DT | ≤0.03 | ≤2/1 | ≤0.5 | ≤0.12 | ≤0.12 | ≤0.5 | ≤0.25 | ≤0.5 | ≤0.25 | ≤0.25 | 1 | ≤0.12 | 1 | ≤1 | 4 | 0.12 | ≤0.5 | ≤1 | 0.03 | ≤0.5/9.5 |
| 71 | 92 | | Yes | B | ≤0.03 | ≤2/1 | ≤0.5 | ≤0.12 | ≤0.12 | ≤0.5 | ≤0.25 | ≤0.5 | ≤0.25 | ≤0.25 | 1 | ≤0.12 | 1 | ≤1 | 4 | ≤0.06 | ≤0.5 | ≤1 | 0.03 | ≤0.5/9.5 |
| 72 | 92 | | No | PS | ≤0.03 | ≤2/1 | ≤0.5 | ≤0.12 | ≤0.12 | ≤0.5 | ≤0.25 | ≤0.5 | ≤0.25 | ≤0.25 | 1 | ≤0.12 | 1 | ≤1 | 4 | ≤0.06 | ≤0.5 | ≤1 | 0.03 | ≤0.5/9.5 |
| 73 | 92 | | No | PS | ≤0.03 | ≤2/1 | ≤0.5 | ≤0.12 | ≤0.12 | ≤0.5 | ≤0.25 | ≤0.5 | ≤0.25 | ≤0.25 | 1 | ≤0.12 | 1 | ≤1 | 4 | ≤0.06 | ≤0.5 | ≤1 | 0.03 | ≤0.5/9.5 |
| 74 | 92 | | No | ASP | ≤0.03 | ≤2/1 | ≤0.5 | ≤0.12 | ≤0.12 | ≤0.5 | ≤0.25 | ≤0.5 | ≤0.25 | ≤0.25 | 1 | ≤0.12 | 1 | ≤1 | 4 | ≤0.06 | ≤0.5 | ≤1 | 0.03 | ≤0.5/9.5 |
| 75 | 92 | | No | PS | ≤0.03 | ≤2/1 | ≤0.5 | ≤0.12 | ≤0.12 | ≤0.5 | ≤0.25 | ≤0.5 | ≤0.25 | ≤0.25 | 1 | ≤0.12 | 1 | ≤1 | 4 | ≤0.06 | ≤0.5 | ≤1 | 0.03 | ≤0.5/9.5 |
| 76 | 76 | | Yes | B | ≤0.03 | ≤2/1 | ≤0.5 | ≤0.12 | ≤0.12 | ≤0.5 | ≤0.25 | ≤0.5 | ≤0.25 | ≤0.25 | 1 | ≤0.12 | 1 | ≤1 | 4 | ≤0.06 | ≤0.5 | >8 | 0.06 | ≤0.5/9.5 |
| 77 | 75 | | No | PS | ≤0.03 | ≤2/1 | ≤0.5 | ≤0.12 | ≤0.12 | 4 | ≤0.25 | ≤0.5 | ≤0.25 | ≤0.25 | 1 | ≤0.12 | 1 | ≤1 | 4 | 0.12 | 1 | ≤1 | 0.03 | ≤0.5/9.5 |
| 78 | 183.2 | | Yes | ASP | ≤0.03 | ≤2/1 | ≤0.5 | ≤0.12 | ≤0.12 | ≤0.5 | ≤0.25 | ≤0.5 | ≤0.25 | ≤0.25 | 1 | ≤0.12 | 1 | ≤1 | 4 | ≤0.06 | ≤0.5 | ≤1 | 0.03 | ≤0.5/9.5 |
| 79 | 183.2 | | No | PS | ≤0.03 | ≤2/1 | ≤0.5 | ≤0.12 | ≤0.12 | ≤0.5 | ≤0.25 | ≤0.5 | ≤0.25 | ≤0.25 | 1 | ≤0.12 | 1 | ≤1 | 4 | 0.12 | ≤0.5 | ≤1 | 0.03 | ≤0.5/9.5 |
| 80 | 183.2 | | Yes | DT | ≤0.03 | ≤2/1 | ≤0.5 | ≤0.12 | ≤0.12 | ≤0.5 | ≤0.25 | ≤0.5 | ≤0.25 | ≤0.25 | 1 | ≤0.12 | 1 | ≤1 | 4 | 0.12 | ≤0.5 | ≤1 | 0.03 | ≤0.5/9.5 |
| 81 | 183.2 | | No | PS | ≤0.03 | ≤2/1 | ≤0.5 | ≤0.12 | ≤0.12 | ≤0.5 | ≤0.25 | ≤0.5 | ≤0.25 | ≤0.25 | 1 | ≤0.12 | 1 | ≤1 | 4 | ≤0.06 | 1 | ≤1 | 0.03 | ≤0.5/9.5 |
| 82 | 184 | | No | PS | ≤0.03 | ≤2/1 | ≤0.5 | ≤0.12 | ≤0.12 | ≤0.5 | ≤0.25 | ≤0.5 | ≤0.25 | ≤0.25 | 1 | ≤0.12 | 1 | ≤1 | 4 | ≤0.06 | ≤0.5 | ≤1 | 0.03 | ≤0.5/9.5 |
| 83 | 184 | | Yes | DT | ≤0.03 | ≤2/1 | ≤0.5 | ≤0.12 | ≤0.12 | ≤0.5 | ≤0.25 | ≤0.5 | ≤0.25 | ≤0.25 | 1 | ≤0.12 | 1 | ≤1 | 4 | ≤0.06 | ≤0.5 | ≤1 | 0.03 | ≤0.5/9.5 |
| 84 | 184 | | No | PS | ≤0.03 | ≤2/1 | ≤0.5 | ≤0.12 | ≤0.12 | ≤0.5 | ≤0.25 | ≤0.5 | ≤0.25 | ≤0.25 | 2 | ≤0.12 | 1 | ≤1 | 4 | ≤0.06 | ≤0.5 | ≤1 | 0.03 | ≤0.5/9.5 |
| 85 | 184 | | Yes | ASP | ≤0.03 | ≤2/1 | ≤0.5 | ≤0.12 | ≤0.12 | ≤0.5 | ≤0.25 | ≤0.5 | ≤0.25 | ≤0.25 | 1 | ≤0.12 | 1 | ≤1 | 4 | ≤0.06 | 1 | ≤1 | 0.03 | ≤0.5/9.5 |
| 86 | 49 | | No | TS | ≤0.03 | ≤2/1 | ≤0.5 | ≤0.12 | ≤0.12 | ≤0.5 | ≤0.25 | ≤0.5 | ≤0.25 | ≤0.25 | 1 | ≤0.12 | 1 | ≤1 | 4 | ≤0.06 | 1 | ≤1 | 0.03 | ≤0.5/9.5 |
| 87 | 49 | | No | TS | ≤0.03 | ≤2/1 | ≤0.5 | ≤0.12 | ≤0.12 | ≤0.5 | ≤0.25 | ≤0.5 | ≤0.25 | ≤0.25 | 1 | ≤0.12 | 1 | ≤1 | 2 | ≤0.06 | ≤0.5 | ≤1 | 0.03 | ≤0.5/9.5 |
| 88 | 44 | | No | TS | ≤0.03 | ≤2/1 | ≤0.5 | ≤0.12 | ≤0.12 | ≤0.5 | ≤0.25 | ≤0.5 | ≤0.25 | ≤0.25 | 1 | ≤0.12 | 1 | ≤1 | 4 | ≤0.06 | ≤0.5 | ≤1 | 0.03 | ≤0.5/9.5 |
| 89 | 44 | | No | TS | ≤0.03 | ≤2/1 | 1 | ≤0.12 | ≤0.12 | ≤0.5 | ≤0.25 | ≤0.5 | ≤0.25 | ≤0.25 | 1 | ≤0.12 | 1 | ≤1 | 4 | ≤0.06 | ≤0.5 | ≤1 | 0.03 | ≤0.5/9.5 |
| 90 | 80 | | No | TS | ≤0.03 | ≤2/1 | ≤0.5 | ≤0.12 | ≤0.12 | ≤0.5 | ≤0.25 | ≤0.5 | ≤0.25 | ≤0.25 | 1 | ≤0.12 | 1 | ≤1 | 4 | ≤0.06 | ≤0.5 | ≤1 | 0.03 | ≤0.5/9.5 |
| 91 | 80 | | No | TS | ≤0.03 | ≤2/1 | ≤0.5 | ≤0.12 | ≤0.12 | ≤0.5 | ≤0.25 | ≤0.5 | ≤0.25 | ≤0.25 | 1 | ≤0.12 | 1 | ≤1 | 4 | ≤0.06 | ≤0.5 | ≤1 | 0.03 | ≤0.5/9.5 |
| 92 | 87 | | No | TS | ≤0.03 | ≤2/1 | ≤0.5 | ≤0.12 | ≤0.12 | ≤0.5 | ≤0.25 | ≤0.5 | ≤0.25 | ≤0.25 | 1 | ≤0.12 | 1 | ≤1 | 4 | 0.12 | ≤0.5 | ≤1 | 0.03 | ≤0.5/9.5 |
| 93 | 87 | | No | TS | ≤0.03 | ≤2/1 | ≤0.5 | ≤0.12 | ≤0.12 | ≤0.5 | ≤0.25 | ≤0.5 | ≤0.25 | ≤0.25 | 1 | ≤0.12 | 1 | ≤1 | 4 | 0.25 | ≤0.5 | ≤1 | 0.03 | ≤0.5/9.5 |
| 94 | 82.1 | | No | TS | ≤0.03 | ≤2/1 | ≤0.5 | ≤0.12 | ≤0.12 | ≤0.5 | ≤0.25 | ≤0.5 | ≤0.25 | ≤0.25 | 1 | ≤0.12 | 1 | ≤1 | 4 | 0.12 | ≤0.5 | ≤1 | 0.03 | ≤0.5/9.5 |
| 95 | 82.1 | | No | TS | ≤0.03 | ≤2/1 | ≤0.5 | ≤0.12 | ≤0.12 | ≤0.5 | ≤0.25 | ≤0.5 | ≤0.25 | ≤0.25 | 1 | ≤0.12 | 1 | ≤1 | 4 | 0.12 | ≤0.5 | ≤1 | 0.06 | ≤0.5/9.5 |
| ET, emmtype; iGAS, Invasive GAS;  PEN, penicillin; AUG2, amoxicillin/clavulanic acid; FUR, cefuroxime; FOT, cefotaxime; AXO, ceftriaxone; FEP, cefepime; MERO, meropenem; ETP, ertapenem; ERY, erythromycin; AZI, azithromycin; LZD, linezolid; CLI, clindamycin; LEVO, levofloxacin; MXF, moxifloxacin; CHL, chloramphenicol; DAP, daptomycin; VAN, vancomycin; TET, tetracycline; TGC, tigecycline; SXT, Sulfamethoxazole/Trimethoprim.  Red, resistant; Orange, intermediate.  PS: pus swab; PA: pus aspirate; ASP: aspirate; AB: abscess; B: blood; DT: deep tissue; TS: throat swab; PF: percutaneous fluid. | | | | | | | | | | | | | | | | | | | | | | | | |

**Table S4. CLSI MIC breakpoints**

| **Antimicrobial agent** | **Susceptible (µg/mL)** | **Intermediate (µg/mL)** | **Resistant (µg/mL)** |
| --- | --- | --- | --- |
| penicillin | ≤0.12 | - | - |
| ampicillin | ≤0.25 | - | - |
| cefuroxime | - | - | - |
| cefotaxime | ≤0.5 | - | - |
| ceftriaxone | ≤0.5 | - | - |
| cefepime | ≤0.5 | - | - |
| meropenem | ≤0.5 | - | - |
| ertapenem | ≤1 | - | - |
| erythromycin | ≤0.25 | 0.5 | ≥1 |
| azithromycin | ≤0.5 | 1 | ≥2 |
| linezolid | ≤2 | - | - |
| clindamycin | ≤0.25 | 0.5 | ≥1 |
| levofloxacin | ≤2 | 4 | ≥8 |
| moxifloxacin | - | - | - |
| chloramphenicol | ≤4 | 8 | ≥16 |
| daptomycin | ≤1 | - | - |
| vancomycin | ≤1 | - | - |
| tetracycline | ≤2 | 4 | ≥8 |
| tigecycline | - | - | - |
| sulfamethoxazole/trimethoprim | - | - | - |
| -, no CLSI breakpoints | | | |
